# Mental Health Care for the Homeless Population: Challenges in a Brazilian Capital

**DOI:** 10.1192/j.eurpsy.2026.11650

**Published:** 2026-07-31

**Authors:** B. L. N. Vercosa, L. P. L. Ferreira, E. R. Nascimento, A. M. D. S. Reinaldo, H. N. Oliveira

**Affiliations:** 1 Faculdade Ciências Medicas de Minas Gerais; 2 Federal University of Minas Gerais, Belo Horizonte, Brazil

## Abstract

**Introduction:**

The increasing number of individuals experiencing homelessness is a global concern, with approximately 2% of the world population living under such conditions. Factors such as unemployment, family conflict, substance use, mental health disorders, and social inequality contribute to this phenomenon. The vulnerability of this population is exacerbated by psychological distress, further complicating the challenges faced by both individuals and health services.

**Objectives:**

To analyze the challenges in providing mental health care to the homeless population in a Brazilian capital, with focus on barriers to access and the quality of care.

**Methods:**

This study employed a qualitative, exploratory approach based on Participatory Action Research. Data collection was conducted through focus groups involving 40 health professionals from a Primary Health Care Center in Belo Horizonte. The data was analyzed using the content analysis methodology proposed by Bardin (2016).

**Results:**

The participants reported several challenges in delivering care, including treatment abandonment, lack of continuity of care, substance use, constant relocation, and difficulty in locating users. These factors highlight significant gaps in the comprehensiveness of the healthcare network, as well as the inadequacy of an effective multidisciplinary approach.

**Conclusions:**

The findings are consistent with both national and international literature, underscoring the urgent need for a multidisciplinary network to meet the mental health demands of this population, which faces both stigma and social invisibility. The absence of an integrated and specialized approach contributes to increased social and psychological vulnerability.

**Disclosure of Interest:**

None Declared

